# Diagnostic Performance of Artificial Intelligence in Predicting Malignant Upgrade of B3 Breast Lesions: Systematic Review and Meta-Analysis

**DOI:** 10.3390/diagnostics16010075

**Published:** 2025-12-25

**Authors:** Romuald Ferre, Cherie M. Kuzmiak

**Affiliations:** Department of Radiology, University of North Carolina at Chapel Hill, Chapel Hill, NC 27599, USA; cherie_kuzmiak@med.unc.edu

**Keywords:** breast imaging, artificial intelligence, B3 lesions, core biopsy, meta-analysis, radiomics, MRI

## Abstract

**Background/Objectives:** High-risk (B3) breast lesions are a heterogeneous group with uncertain malignant potential. **Methods:** We systematically reviewed and meta-analyzed the ability of artificial-intelligence (AI) models to predict malignant upgrades (a ductal carcinoma in situ or an invasive carcinoma) after biopsy. A comprehensive search of medical and engineering databases through 27 July 2025 identified retrospective studies that developed or validated AI models for upgrade prediction in cohorts with ≥20 B3 lesions and confirmed outcomes at surgical excision or after ≥24 months of follow-up. **Results:** Three single-center studies (557 lesions, 91 upgrades) met the eligibility criteria. Pooled analysis focused on clinically meaningful operating points rather than raw accuracy metrics. Models tuned for high sensitivity achieved high negative predictive values (pooled 0.95), suggesting reliable identification of lesions suitable for surveillance, but positive predictive values were modest and heterogenous (0.15–1.00), reflecting trade-offs between avoiding missed upgrades and reducing unnecessary excisions. Only two studies reported area-under-the-receiver-operating-characteristic curves, which pooled to 0.72, indicating moderate discrimination. **Conclusions:** Although limited by small sample sizes and single-center designs, these findings suggest that AI could aid decision-making for B3 lesion management. Prospective multicenter validation and standardized reporting are needed to evaluate clinical utility.

## 1. Introduction

High-risk (B3) breast lesions are a frequent outcome of image-guided core or vacuum-assisted breast biopsy and present a persistent management dilemma. B3 lesions comprise a heterogeneous group of pathologies—including atypical ductal hyperplasia (ADH), lobular neoplasia, flat epithelial atypia, radial scars/complex sclerosing lesions, papillary lesions, and selected fibroepithelial lesions—that share uncertain malignant potential rather than uniform biological behavior [1,2,3,4]. Many series and meta-analyses report overall malignant upgrade rates of roughly 10–35% at surgical excision, but risks vary substantially by lesion subtype, radiologic–pathologic concordance, biopsy method, and sampling adequacy [2].

Over the past decade, consensus conferences and guidelines have moved from a “one-size-fits-all” excision policy toward more individualized, risk-adapted management. The Second and Third International Consensus Conferences on B3 lesions and national guidelines, including those from European working groups and the American Society of Breast Surgeons, recommended that decisions about surgical excision versus imaging surveillance or minimally invasive excision be based on lesion subtype, radiologic–pathologic concordance, biopsy method, patient risk factors, and preferences [5,6]. Large multicenter cohorts and long-term follow-up studies have confirmed that both the upgrade risk and the safety of non-operative management depend heavily on lesion-level and patient-level factors [7].

In parallel, artificial intelligence (AI) and radiomics have been widely investigated in breast imaging [8,9,10,11,12]. Radiomics enables extraction of quantitative imaging features that capture shape, intensity, and texture patterns, which can be combined with clinical and pathological data in machine learning (ML) or deep learning (DL) models to generate individualized risk predictions. In broader breast imaging tasks, AI models have achieved high discrimination for lesion classification, molecular subtype prediction, and treatment response assessment across MRI, ultrasound, and mammography or digital breast tomosynthesis, although external validation results and real-world impacts remain variable.

Several groups have applied these techniques specifically to high-risk and B3 lesions. For example, ML models using clinical and imaging variables have been developed to predict pathologic upgrades and potentially reduce unnecessary excisions [10,11] However, the existing literature remains fragmented: published studies differ in terms of lesion spectra, imaging protocols, AI architectures, and validation strategies, and many are single-center retrospective cohorts with modest sample sizes and primarily internal validation. Reporting of model development and performance is also heterogeneous, despite the availability of tools such as PROBAST and TRIPOD-AI [13,14].

Consequently, clinicians and methodologists lack a consolidated, quantitative assessment of how well AI currently performs for predicting malignant upgrades of B3 lesions and what range of performance might be expected in new settings. We therefore conducted a systematic review and meta-analysis to (i) summarize the diagnostic performance of AI models in predicting malignant upgrades in B3 breast lesions; (ii) quantify between-study heterogeneity and the expected dispersion of true effects; and (iii) explore potential sources of variability, including imaging modality and validation strategy. Our goal is to provide a decision-relevant synthesis to guide further method development, reporting standards, and prospective evaluation in clinical workflows.

## 2. Materials and Methods

This systematic review followed PRISMA 2020 (Table S1) and was conducted according to a prespecified protocol registered in PROSPERO (registration number: CRD420251250934). Because only published, aggregate-level data were used, institutional review board approval was not required. We appraised prediction-model design and reporting using PROBAST and interpreted findings with reference to TRIPOD + AI guidance.

### 2.1. Data Sources and Search Strategy

With the assistance of an information specialist, we searched MEDLINE, Embase, Scopus, Web of Science, CENTRAL, IEEE Xplore, arXiv, and medRxiv from June to December 2025. Search concepts combined (i) B3/high-risk lesions (e.g., “B3 lesion,” “high-risk lesion,” “atypical ductal hyperplasia,” “lobular neoplasia,” “flat epithelial atypia,” “papilloma,” and “radial scar”); (ii) upgrade/underestimation outcomes (e.g., “upgrade,” “underestimation,” “pathologic upgrade,” and “malignant upgrade”); and (iii) AI/ML terms (e.g., “machine learning,” “artificial intelligence,” “radiomics,” “deep learning,” “neural network,” and “support vector machine”). We screened reference lists of eligible studies and relevant reviews/consensus statements.

No restrictions were placed on publication year. We screened conference abstracts and gray literature at title/abstract level to maximize sensitivity; when an abstract appeared eligible but lacked a full manuscript, we searched for corresponding preprints or full publications. Only human studies were included. Non-English full texts were excluded if reliable extraction/translation was not feasible.

### 2.2. Eligibility Criteria

We included studies meeting all the following criteria:

1. Population: Patients with biopsy-proven high-risk (B3 or equivalent) breast lesions diagnosed via core-needle or vacuum-assisted biopsy.
2. Index model: Development or validation of an AI/ML model intended to predict malignant upgrades (DCIS or invasive carcinoma) upon making a surgical excision and/or malignant outcomes at a follow-up. Models could use imaging-derived inputs, conventional radiologic descriptors, clinical variables, pathological variables, or combinations.
3. Sample size: ≥20 high-risk/B3 lesions.
4. Reference standard: Surgical pathology or ≥24-month imaging follow-up for lesions not excised.
5. Outcomes: Reported or derivable diagnostic-performance data. For quantitative pooling of predictive values, studies had to provide enough information at a stated operating point (threshold) to allow derivation of a 2 × 2 table (TP/FP/TN/FN) or directly report PPV/NPV with denominators.

We excluded case reports, editorials/letters, narrative reviews, animal/phantom studies, and “technical-only” AI papers without clinical outcome validation. Studies analyzing mixed cohorts without B3/high-risk subgroup performance or predicting long-term breast cancer risk rather than near-term upgrades during excision/follow-up, were excluded.

### 2.3. Study Selection and Data Extraction

Two reviewers independently screened titles/abstracts and then full texts. Discrepancies were resolved by discussion and, if needed, senior adjudication. Full-text exclusion reasons were recorded.

Two reviewers independently extracted the following data:

- Study characteristics: country, design, enrollment period, setting, inclusion criteria.
- Cohort details: number of lesions, lesion subtype mix (e.g., ADH vs. mixed B3), biopsy method, and upgrade prevalence.
- Model details: predictors used (pathological, descriptors and imaging-derived features), algorithm used (random forest, SVM, etc.), and validation approach.
- Operating point: how the threshold was chosen (e.g., fixed predicted-risk cut-off or sensitivity-targeted cut-off).
- Performance: sensitivity, specificity, AUC (if reported), and predictive values (PPV/NPV) [15,16].

When studies reported multiple models/thresholds, we extracted the model/threshold emphasized for clinical decision-making (typically the primary model or the operating point linked to excision-versus-surveillance recommendations).

### 2.4. Risk of Bias and Applicability

Risk of bias and applicability were assessed with PROBAST (participants, predictors, outcome, and analysis). Each domain was rated as low, high, or unclear, with an overall high-risk judgement if any domain was high risk. Two reviewers assessed cases independently and resolved disagreements by consensus.

### 2.5. Statistical Analysis

Because the management decision for B3/high-risk lesions is typically excision versus surveillance, we treated model output as a binary triage at each study’s reported operating point:

- Test positive: model recommends excision/“high risk”.
- Test negative: model supports surveillance/“low risk”.
- From each study, we derived (or extracted) TP/FP/TN/FN counts at the stated operating point and calculated the following:
- PPV—upgraded cancers among predicted-excision lesions (surgical yield).
- NPV—non-upgraded lesions among predicted-surveillance lesions (rule-out reassurance).

We computed 95% confidence intervals for PPV/NPV using binomial methods. We pooled PPV and NPV across studies using random-effects meta-analysis of proportions on the logit scale, reporting pooled estimates with 95% CIs and heterogeneity statistics (I^2^, τ^2^). Given the small number of studies and differing threshold choices, pooled predictive values were interpreted as descriptive summaries rather than definitive generalizable effects [17,18,19,20,21].

For AUC, we extracted reported AUCs and (when needed) derived standard errors from available information. AUC pooling was performed only when at least two studies reported extractable AUCs. Sensitivity and specificity were summarized descriptively because operating points differed substantially between studies.

## 3. Results

### 3.1. Study Selection

**Figure 1:**
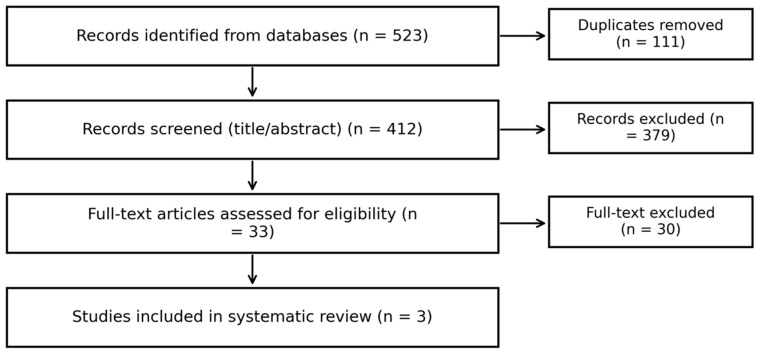
Prisma flow chart.

Three studies met the inclusion criteria for the quantitative synthesis of predictive values (Bahl, Harrington, Aslan), comprising 557 lesions and 91 malignant upgrades overall (Figure 1).

### 3.2. Study Characteristics

All included studies were retrospective single-center cohorts published between 2017 and 2023. The clinical scope differed across studies: one cohort focused on ADH only, whereas others included a broader high-risk/B3 spectrum. Models also differed in terms of inputs and intended clinical operating points:

- Bahl [22]: a machine-learning model using structured clinical/imaging-pathology variables (and report-derived features) with a low risk threshold (e.g., >5%) intended to prioritize sensitivity.
- Harrington [23]: An ML model for ADH upgrades, reported at an operating point targeting very high sensitivity.
- Aslan [24]: ML classifiers using clinical and radiologic descriptors. The selected SVM operating point emphasized specificity, producing a very low false-positive rate.

Upgrade prevalence ranged from ~11% to ~25%, contributing to between-study variability in predictive values (Table 1, Table 2 and Table 3).

### 3.3. Risk of Bias and Applicability

Using PROBAST, we deemed two of the three included studies to have an overall high risk of bias, driven primarily by limitations in the analysis domain. Harrington et al. developed a random-forest model using nested cross-validation for 128 ADH lesions (30 upgrades), but the combination of limited event numbers relative to model complexity (32 features) and the absence of external validation resulted in a high risk-of-bias judgement.

Aslan et al. used an 80/20 split (75 training, 19 testing) for a dataset of 94 patients (23 malignant upgrades); the small test set and partial reliance on follow-ups rather than excision for benign outcomes contributed to a high risk of bias and applicability concerns.

Bahl et al. used a larger cohort with an independent test set and defined upgrade as DCIS or invasive carcinoma determined upon excision, but several key analysis details (e.g., fully transparent handling of high-dimensional predictors and other reporting elements needed for PROBAST) were insufficiently extractable; therefore, the overall risk of bias was judged to be unclear.

### 3.4. Predictive Performance at Study-Selected Operating Points

Across studies, sensitivity ranged from 0.61 to 0.98, and specificity ranged from 0.16 to 1.00, reflecting markedly different threshold choices and clinical priorities.

At the stated operating points,

- PPV ranged from 0.15 to 1.00;
- NPV ranged from 0.89 to 0.99.

Importantly, the same “good-looking” PPV/NPV values corresponded to very different clinical behaviors:

- In high-sensitivity settings (Bahl; Harrington), NPV was high, but specificity was low and the model recommended excision for most lesions (high false-positive burden).
- In the high-specificity setting (Aslan), PPV was very high, but sensitivity was substantially lower, with more missed upgrades.

### 3.5. Meta-Analysis of PPV

Pooling the three studies yielded a pooled PPV of 0.291 (95% CI 0.128–0.533) with substantial heterogeneity (I^2^ 88%). This heterogeneity is to be expected because PPV is strongly influenced by (i) upgrade prevalence and (ii) the aggressiveness of the operating threshold.

**Figure 2:**
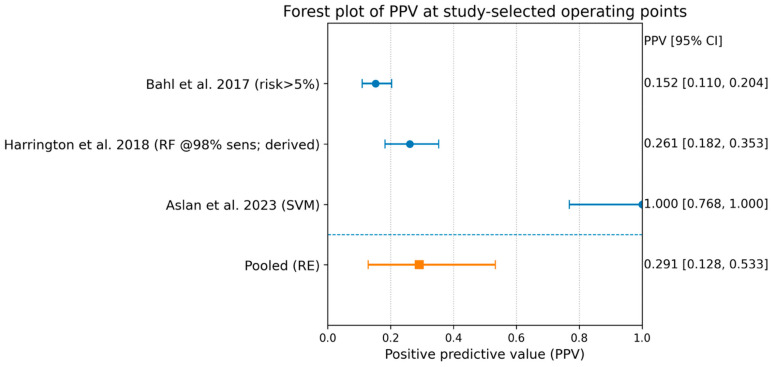
Forest plot of PPV [22,23,24].

A sensitivity analysis excluding the high-specificity outlier pattern (Aslan) reduced the pooled PPV to 0.200 (95% CI 0.114–0.326), but heterogeneity remained high (Figure 2).

### 3.6. Meta-Analysis of NPV

**Figure 3:**
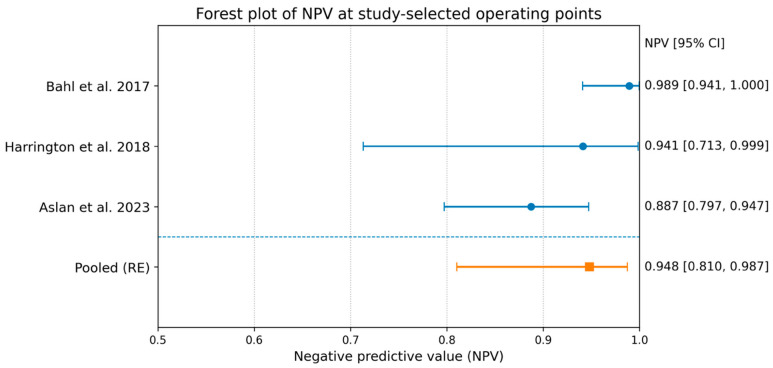
Forest plot of NPV [22,23,24].

Pooling the three studies yielded a pooled NPV of 0.948 (95% CI 0.810–0.987) with moderate heterogeneity (I^2^ 63%). Excluding Aslan increased the pooled NPV to 0.975 (95% CI 0.876–0.995), which is consistent with the higher-sensitivity operating points used in the remaining studies (Figure 3).

### 3.7. AUC

Only two studies reported extractable AUCs (Harrington; Aslan). The pooled AUC across these two studies was 0.722 (95% CI 0.595–0.822) with moderate heterogeneity (I^2^ 42%). Because AUC was not consistently reported across all included studies (and because thresholds differed), the AUC results should be interpreted as supporting context rather than the sole determinant of clinical utility.

## 4. Discussion

### 4.1. Principal Findings

In this focused synthesis of three retrospective, single-center machine-learning (ML) studies (Bahl, Harrington, Aslan), encompassing 557 lesions and 91 malignant upgrades, predictive values suggested a consistent “rule-out” signal but marked threshold-dependent trade-offs. Specifically, the pooled negative predictive value (NPV) was 0.948 (95% CI 0.810–0.987), whereas the pooled positive predictive value (PPV) was 0.291 (95% CI 0.128–0.533) with substantial heterogeneity (I^2^ 88%). Where AUC was extractable (two studies), discrimination was moderate (pooled AUC 0.722, 95% CI 0.595–0.822).

Crucially, the headline finding is not simply that “NPV is high,” but that *similar-looking* NPVs were achieved through very different, and clinically consequential, operating points. Across studies, sensitivity ranged from 0.61 to 0.98, and specificity ranged from 0.16 to 1.00, reflecting divergent threshold choices and clinical priorities. In the high-sensitivity approaches (Bahl; Harrington), models prioritized avoiding missed upgrades at the expense of many false positives (i.e., recommending excision for most lesions).

By contrast, Aslan’s selected operating point emphasized specificity (a very low false-positive rate), resulting in a “high-yield” excision recommendation among those labeled positive, but with a larger missed-upgrade burden.

These study-level patterns are consistent with how the underlying models were framed clinically [25]. For example, Bahl et al. reported that their ML strategy could avoid 30.6% of surgeries for benign lesions (91/297) while maintaining cancer detection, and in one comparison, it diagnosed 97.4% of cancers (37/38).

Harrington et al. [23] (ADH-only cohort) reported best AUCs of ~0.68 and described a high-sensitivity operating point in which 98% of malignancies would be diagnosed while 16% of unnecessary surgeries could be avoided (87% sensitivity, 45% specificity). Aslan et al. [24] reported an upgrade prevalence of 24.5% and an SVM model with an AUC of 0.786 and 0.84 accuracy.

### 4.2. Should NPV Be Emphasized?

If the intended clinical use is *to safely defer surgery* (i.e., “rule out” upgrade and support surveillance), then the NPV is an appropriate headline metric—but it must be presented together with the false-negative rate (or sensitivity) and clear context, because the NPV is highly dependent on (i) upgrade prevalence in the target population, (ii) threshold choice, and (iii) verification strategy (who undergoes excision versus surveillance and for how long).

A decision-focused framing that addresses interpretability and heterogeneity can therefore be defined as follows:

- Primary (safety): sensitivity/missed-upgrade rate plus NPV (confidence in surveillance recommendations);
- Secondary (burden/yield): PPV plus the implied excision rate (how many patients the model would send to surgery);
- Contextual (threshold-free): AUC (and calibration, if available).

This is especially important here because the pooled NPV (0.948) increased to 0.975 when the high-specificity operating-point pattern was excluded, illustrating how a strongly pooled NPV can shift when the decision threshold strategy changes.

### 4.3. Why PPV Was So Variable

The large heterogeneity in PPVs is to be expected, and it is clinically informative rather than “noise.” In this review, PPVs ranged from 0.15 to 1.00, and pooled PPVs showed substantial heterogeneity (I^2^ 88%).

The PPV rises when either (a) the underlying upgrade prevalence is higher or (b) the operating threshold is set such that it will reduce false positives (higher specificity).

However, shifting toward high specificity typically reduces sensitivity and increases missed upgrades—often an unacceptable trade-off if the primary goal is to *avoid missing carcinoma* in lesions triaged to surveillance.

Accordingly, PPV should be interpreted as a measure of *surgical yield at a chosen operating point*, not a fixed property of the model.

### 4.4. Clinical Implications

Taken together, the current evidence supports using ML models primarily as decision-support tools for de-escalation, but only if a “surveillance/low-risk” recommendation is shown—preferably in external validation and ideally prospectively—to correspond to an acceptably low residual upgrade risk in the intended population.

To make model performance clinically interpretable and transportable across practice settings, future B3/high-risk upgrade studies (and systematic syntheses) should consistently report

- The excision rate implied by the chosen threshold;
- The missed-upgrade count and proportion (false negatives);
- Calibration (so predicted probabilities reflect observed risks);
- Clinical-utility analyses such as decision-curve analysis across plausible threshold ranges.

### 4.5. Limitations

This synthesis is constrained by the small evidence base (k = 3) and the fact that the studies included were retrospective and single-center.

Interpretation is further limited by non-comparable operating points (thresholds were chosen for different clinical priorities), variation in the case mix (e.g., ADH-only versus broader B3/high-risk cohorts), and inconsistent reporting of performance metrics (the AUC was not extractable for all studies, and there was limited calibration reporting).

For these reasons, pooled PPVs/NPVs should be treated as descriptive summaries of plausible clinical trade-offs, not as definitive performance targets for deployment.

## 5. Conclusions

Across three ML studies of upgrade prediction for high-risk/B3 lesions, the NPV is generally high (pooled ~0.95), but its meaning depends strongly on prevalence, verification, and—most importantly—threshold choice.

PPVs are modest on average and highly heterogeneous, reflecting different operating-point strategies rather than a uniform “model effect.”

Where available, AUC values suggest moderate discrimination, but the AUC alone cannot resolve the threshold-dependent clinical trade-offs that determine safety (missed upgrades) and burden (excision rate).

## Figures and Tables

**Table 1:** Study comparison.

Study	Target Population	Total Lesions (*n*)	Upgraded to Cancer, *n* (%)	Non-Upgrade (*n*)
Bahl et al. (2017) [22]	High-risk breast lesions (HRLs) found via image-guided core biopsy	1006	115 (11.4%)	891
Harrington et al. (2018) [23]	Atypical ductal hyperplasia (ADH) found via core needle biopsy with surgical excision outcomes	128	30 (23.4%)	98
Aslan et al. (2023) [24]	High-risk breast lesions (HRLs), mixed subtypes	94	23 (24.5%)	71

**Table 2:** Data for each study.

Study	Model	Sensitivity	Specificity	PPV	NPV
Bahl et al. [22]	Random forest	0.97 (37/38)	0.31 (91/297)	0.15 (37/243)	0.99 (91/92)
Harrington et al. [23]	Random forest	0.98	0.16	0.26	0.96
Aslan et al. [24]	SVM	0.61	1.00	1.00	0.89

**Table 3:** Meta-analysis.

Study	True Negatives in Predicted Surveillance (TN)	Total Predicted Surveillance (TN + FN)	NPV	95% CI (Lower)	95% CI (Upper)	
Bahl et al. (2017) [22]	91	92	0.989	0.941	1.000	
Harrington et al. (2018) [23]	16	17	0.941	0.713	0.999	
Aslan et al. (2023) [24]	71	80	0.888	0.797	0.947	
**Summary**	**k**	**Pooled NPV**	**95% CI (Lower)**	**95% CI (Upper)**	**I^2^ (%)**	**τ^2^**
Random-effects pooled NPV (AI-only; Bahl + Harrington + Aslan)	3	0.948	0.810	0.987	63.1	1.039

## Data Availability

Not applicable.

## Supplementary Materials

The following supporting information can be downloaded at: https://www.mdpi.com/article/10.3390/diagnostics16010075/s1, Table S1. PRISMA 2020 Checklist for the Systematic Review.
