# Supplementary material for: Diagnostic Performance of Artificial Intelligence in Predicting Malignant Upgrade of B3 Breast Lesions: Systematic Review and Meta-Analysis
Source: Diagnostics (Basel). 2025 Dec 25;16(1):75. doi: 10.3390/diagnostics16010075 (PMC12785790; doi:10.3390/diagnostics16010075)
Supplement: Supplementary file 1 [file diagnostics-16-00075-s001.zip › diagnostics-4031689-supplementary.pdf]

Table S1. PRISMA 2020 Checklist for the Systematic Review

Source: PRISMA 2020 checklist (CC BY 4.0). https://www.prisma-statement.org/prisma-2020-checklist

Section	Topic	Item #	Checklist item	Location where item is reported
TITLE	Title	1	Identify the report as a systematic review.	p.1 (Title)
ABSTRACT	Abstract	2	See the PRISMA 2020 for Abstracts checklist.	p.1 (Abstract)
INTRODUCTION	Rationale	3	Describe the rationale for the review in the context of existing knowledge.	pp.2–3 (1. Introduction)
INTRODUCTION	Objectives	4	Provide an explicit statement of the objective(s) or question(s) the review addresses.	p.3 (1. Introduction—Objectives paragraph)
METHODS	Eligibility criteria	5	Specify the inclusion and exclusion criteria for the review and how studies were grouped for the syntheses.	p.4 (2.2 Eligibility Criteria)
METHODS	Information sources	6	Specify all databases, registers, websites, organisations, reference lists and other sources searched or consulted to identify studies. Specify the date when each source was last searched or consulted.	p.1 (Abstract—Methods); p.3 (2.1 Data Sources and Search Strategy)
METHODS	Search strategy	7	Present the full search strategies for all databases, registers and websites, including any filters and limits used.	p.3 (2.1 Search Strategy—concepts/keywords described; full search strings not reported)
METHODS	Selection process	8	Specify the methods used to decide whether a study met the inclusion criteria of the review, including how many reviewers screened each record and each report retrieved, whether they worked independently, and if applicable, details of automation tools used in the process.	p.4 (2.3 Study Selection and Data Extraction)
METHODS	Data collection process	9	Specify the methods used to collect data from reports, including how many reviewers collected data from each report, whether they worked independently, any processes for obtaining or confirming data from study investigators, and if applicable, details of automation tools used in the process.	p.4 (2.3 Study Selection and Data Extraction)

METHODS	Data items	10a	List and define all outcomes for which data were sought. Specify whether all results that were compatible with each outcome domain in each study were sought (e.g. for all measures, time points, analyses), and if not, the methods used to decide which results to collect.	p.4 (2.3); p.6 (2.5 Statistical Analysis); Tables 2–3 (p.8)
METHODS	Data items	10b	List and define all other variables for which data were sought (e.g. participant and intervention characteristics, funding sources). Describe any assumptions made about any missing or unclear information.	p.4 (2.3); Table 1 (p.7)
METHODS	Study risk of bias assessment	11	Specify the methods used to assess risk of bias in the included studies, including details of the tool(s) used, how many reviewers assessed each study and whether they worked independently, and if applicable, details of automation tools used in the process.	p.6 (2.4 Risk of Bias and Applicability); p.8 (3.3 Risk of Bias and Applicability)
METHODS	Effect measures	12	Specify for each outcome the effect measure(s) (e.g. risk ratio, mean difference) used in the synthesis or presentation of results.	p.6 (2.5 Statistical Analysis)
METHODS	Synthesis methods	13a	Describe the processes used to decide which studies were eligible for each synthesis (e.g. tabulating the study intervention characteristics and comparing against the planned groups for each synthesis (item #5)).	p.4 (2.2 Eligibility Criteria—data required for 2×2); p.6 (2.5 Statistical Analysis—criteria for pooling PPV/NPV and AUC)
METHODS	Synthesis methods	13b	Describe any methods required to prepare the data for presentation or synthesis, such as handling of missing summary statistics, or data conversions.	p.6 (2.5 Statistical Analysis—derive TP/FP/TN/FN; compute CIs; derive AUC SEs when needed)
METHODS	Synthesis methods	13c	Describe any methods used to tabulate or visually display results of individual studies and syntheses.	Figure 1 (pp.6–7); Tables 1–3 (pp.7–8); Figures 2–3 (pp.9–11)
METHODS	Synthesis methods	13d	Describe any methods used to synthesize results and provide a rationale for the choice(s). If meta-analysis was performed,	p.6 (2.5 Statistical Analysis—random-effects meta-analysis; I^2, τ^2)

			describe the model(s), method(s) to identify the presence and extent of statistical heterogeneity, and software package(s) used.	
METHODS	Synthesis methods	13e	Describe any methods used to explore possible causes of heterogeneity among study results (e.g. subgroup analysis, meta-regression).	p.6 (2.5 Statistical Analysis—heterogeneity statistics); Results 3.5–3.7 (pp.9–11; I^2/τ^2 reported)
METHODS	Synthesis methods	13f	Describe any sensitivity analyses conducted to assess robustness of the synthesized results.	p.9 and p.11 (Results 3.5–3.6 sensitivity analyses excluding Aslan)
METHODS	Reporting bias assessment	14	Describe any methods used to assess risk of bias due to missing results in a synthesis (arising from reporting biases).	P4
METHODS	Certainty assessment	15	Describe any methods used to assess certainty (or confidence) in the body of evidence for an outcome.	P4
RESULTS	Study selection	16a	Describe the results of the search and selection process, from the number of records identified in the search to the number of studies included in the review, ideally using a flow diagram.	p.6 (3.1 Study Selection); Figure 1 (pp.6–7)
RESULTS	Study selection	16b	Cite studies that might appear to meet the inclusion criteria, but which were excluded, and explain why they were excluded.	Not reported (full-text exclusions/reasons not presented)
RESULTS	Study characteristics	17	Cite each included study and present its characteristics.	p.7 (3.2 Study Characteristics); Tables 1–2 (pp.7–8)
RESULTS	Risk of bias in studies	18	Present assessments of risk of bias for each included study.	p.8 (3.3 Risk of Bias and Applicability)
RESULTS	Results of individual studies	19	For all outcomes, present, for each study: (a) summary statistics for each group (where appropriate) and (b) an effect estimate and its precision (e.g. confidence/credible interval), ideally using structured tables or plots.	Tables 2–3 (p.8); Results 3.4 (p.9); AUC results 3.7 (p.11)
RESULTS	Results of syntheses	20a	For each synthesis, briefly summarise the characteristics and risk of bias among contributing studies.	p.9 (3.4 Predictive Performance); Tables 2–3

RESULTS	Results of syntheses	20b	Present results of all statistical syntheses conducted. If meta-analysis was done, present for each the summary estimate and its precision (e.g. confidence/credible interval) and measures of statistical heterogeneity. If comparing groups, describe the direction of the effect.	Results 3.5–3.7 (pp.9–11); Figures 2–3 (pp.9–11)
RESULTS	Results of syntheses	20c	Present results of all investigations of possible causes of heterogeneity among study results.	Results 3.5–3.7 (pp.9–11; I^2/τ^2 reported)
RESULTS	Results of syntheses	20d	Present results of all sensitivity analyses conducted to assess the robustness of the synthesized results.	p.9 and p.11 (sensitivity analyses excluding Aslan)
RESULTS	Reporting biases	21	Present assessments of risk of bias due to missing results (arising from reporting biases) for each synthesis assessed.	P6
RESULTS	Certainty of evidence	22	Present assessments of certainty (or confidence) in the body of evidence for each outcome assessed.	P7
DISCUSSION	Discussion	23a	Provide a general interpretation of the results in the context of other evidence.	Discussion 4.1–4.4 (pp.11–13); Conclusions (p.14)
DISCUSSION	Discussion	23b	Discuss any limitations of the evidence included in the review.	Limitations 4.5 (p.13)
DISCUSSION	Discussion	23c	Discuss any limitations of the review processes used.	Not explicitly discussed (limitations of review processes)
DISCUSSION	Discussion	23d	Discuss implications of the results for practice, policy, and future research.	Clinical implications 4.4 (p.13); Conclusions (p.14)
OTHER INFORMATION	Registration and protocol	24a	Provide registration information for the review, including register name and registration number, or state that the review was not registered.	p.3 (2. Materials and Methods—PROSPERO registration CRD420251250934)
OTHER INFORMATION	Registration and protocol	24b	Indicate where the review protocol can be accessed, or state that a protocol was not prepared.	p.3 (protocol registered in PROSPERO; protocol access not otherwise stated)
OTHER INFORMATION	Registration and protocol	24c	Describe and explain any amendments to information provided at registration or in the protocol.	Not reported (no protocol amendments described)

OTHER INFORMATION	Support	25	Describe sources of financial or non-financial support for the review, and the role of the funders or sponsors in the review.	p.14 (Funding statement); p.3 (2.1 information specialist assistance)
OTHER INFORMATION	Competing interests	26	Declare any competing interests of review authors.	p.14 (Conflicts of Interest)
OTHER INFORMATION	Availability of data, code and other materials	27	Report which of the following are publicly available and where they can be found: template data collection forms; data extracted from included studies; data used for all analyses; analytic code; any other materials used in the review.	p.14 (Data Availability Statement; Supplementary Materials—Table S1)
